# Perivascular space imaging during therapy for medulloblastoma

**DOI:** 10.1371/journal.pone.0318278

**Published:** 2025-02-07

**Authors:** Ruitian Song, John O. Glass, Shengjie Wu, Yimei Li, Giles W. Robinson, Amar Gajjar, Thomas E. Merchant, Wilburn E. Reddick

**Affiliations:** 1 Diagnostic Imaging, St Jude Children’s Research Hospital, Memphis, Tennessee, United States of America; 2 Biostatistics, St Jude Children’s Research Hospital, Memphis, Tennessee, United States of America; 3 Oncology, St Jude Children’s Research Hospital, Memphis, Tennessee, United States of America; 4 Pediatric Medicine, St Jude Children’s Research Hospital, Memphis, Tennessee, United States of America; 5 Radiation Oncology, St Jude Children’s Research Hospital, Memphis, Tennessee, United States of America; Memorial Sloan Kettering Cancer Center, UNITED STATES OF AMERICA

## Abstract

Perivascular spaces (PVS) are fluid filled compartments surrounding the small blood vessels in the brain. The impact of radiotherapy and chemotherapy on PVS remains unclear. The aim of this study is to investigate treatment effects of radiotherapy and chemotherapy at four time points (TPs) in pediatric medulloblastoma (MB) patients. We examined 778 scans from 241 MB patients at baseline (0M), after 12 weeks (about 3 months) of radiotherapy and rest (3M), after chemotherapy completion (12M), and a follow-up (FollowUp) at 18- or 21-months post-baseline. PVS was segmented by applying Frangi filter on the white matter regions on T1 weighted images acquired at 3T Siemens MRI scanner using MPRAGE. PVS volume and ratio, defined as the ratio of PVS volume to the white matter volume, were measured at the four TPs. The data was first statistically analyzed using a full model where all data were included, then a paired model, which included only patients who completed consecutive measurements under the same anesthesia and shunt conditions. Both the full model and paired model showed that PVS (including ratio and volume) increased at 3M post-radiotherapy compared to baseline. During chemotherapy, PVS decreased significantly from 3M to 12M. Subsequently, from 12M to FollowUp, PVS increased again. MRI exams under anesthesia exhibited significantly lower PVS than those without anesthesia. Patients who had undergone a shunt procedure exhibited a significantly reduced PVS compared to those who had not undergone the procedure. We concluded that craniospinal irradiation led to an elevated PVS. Conversely, chemotherapy or time post-irradiation decreased PVS. Anesthesia and shunt procedures can also influence perivascular space ratio or volume.

## Introduction

Medulloblastoma (MB) is a malignant brain tumor originating in the cerebellum, with the potential to metastasize through cerebrospinal fluid (CSF) pathways to other regions involving the brain and spinal cord. MB accounts for approximately 20% of all brain tumors and is relatively rare in adults [1]. Treatment typically involves surgery followed by craniospinal irradiation and post-irradiation systemic chemotherapy [2]. Advances in therapy have improved the long-term survival rates to more than 80% for low- and standard-risk patients [3]. However, a substantial portion of patients, especially those with high-risk presentations, do not survive, or suffer complications after curative combined modality therapy [3].

The glymphatic system utilizes perivascular spaces (PVS) and astrocytic water channels for waste clearance. PVS, also known as Virchow-Robin spaces, are fluid-filled compartments surrounding small blood vessels in the brain and play a crucial role in the brain’s glymphatic clearance system [4]. Recent studies have suggested that enlarged PVS may be an indicator of increased risk of stroke [5], and have also been observed in a range of neurological disorders [4,6].

MRI is a powerful tool to investigate PVS. The signal intensity of PVS matches that of CSF. PVS appear as tube-like hypointense on a T1 weighted (T1w) image or hyperintense on a T2 weighted (T2w) images. Traditionally PVS is graded according to established visual rating scales [7,8], which provide a qualitative estimate of the extent of PVS burden. However, manual counting lacks precision and visual rating scales also suffer from low sensitivity [8]. Recent advances in image processing technology has enabled automatic segmentation using the marked point process framework [9], vesselness filters [10,11], intensity-based thresholding [12,13], and machine learning techniques [14,15]. The PVS segmentation has been applied to T1w images [9,16,17], proton density images [18], T2w images [6,19], or combinations of T1w and T2w images [11,20]. Diffusion tensor image analysis along the perivascular space (DTI-ALPS) index [21] is another tool to assess glymphatic clearance. It distinguishes the contributions of projection, association, and subcortical fibers. The diffusion values in these three directions are then combined arithmetically to quantify diffusion along the direction of the perivascular space. The DTI-ALPS index is negatively correlated with the PVS volume or ratio, and they often play reversed roles for diseased states [22,23].

The mechanisms underlying PVS dilation remains incompletely understood, but several explanations have been proposed, including increased arterial wall permeability, increased permeability of the blood-brain barrier(BBB), alterations in CSF drainage, spiral elongation of blood vessels, or inflammatory activity in the brain [24–26]. Interestingly, some of these changes share characteristics with pathological responses seen after radiotherapy, including white matter edema, demyelination, and fibrinoid changes in blood vessels [27]. These similarities raise the important hypothesis that radiotherapy may impact PVS in a similar way. Although a couple of clinical findings have previously reported a potential association between enlarged PVS and radiotherapy, such observations were limited to one or two isolated clinical cases, lacking robust statistical conclusions [28,29]. Furthermore, the effects of chemotherapy on PVS remain largely unexplored.

Aside from pathological factors, clinical variables may influence PVS, including common practices such as anesthesia in examining pediatric patients and the shunt CSF diversion procedures employed in the treatment of MB patients. However, the specific impacts of these practices on PVS measurements require further investigation. Considering the significant clinical value of PVS in the evaluation of various neurological disorders [30,31], it is essential to gain a clear understanding of the effects of anesthesia and shunt CSF diversion procedures on these measures before utilizing them as tools to investigate neurological conditions.

In this study, we investigated 778 exams from 241 patients with MB received craniospinal irradiation and chemotherapy. PVS were measured at four distinct stages of treatment: baseline, after radiotherapy and rest, after completion of chemotherapy, and follow up. The main objective of the study was to assess the impact of radiotherapy and chemotherapy on PVS. Additionally, we investigated the effect of anesthesia and CSF shunt procedures on the PVS ratio.

## Materials and methods

The study cohort consisted of patients who participated in a clinical trial for newly diagnosed medulloblastoma (NCT01878617, https://clinicaltrials.gov/study/NCT01878617) conducted from June 2013 to April 2023. No patients had received radiotherapy or chemotherapy prior to the study. All patients had completed resection surgery treatments to remove the tumors before the baseline. Therapy was based on the molecular and risk group of participants. Three molecular subgroups were defined as WNT, SHH, and non-WNT/ non-SHH. Risk stratification was defined based on metastases, degree of resection, and histology (Classic, MONO6, MYC/MYCN amplified). All participants underwent risk-adapted craniospinal irradiation (CSI) with a boost to the primary tumor site. The CSI dose given was one of three levels: reduced dose (RD) of 15Gy, standard dose (SD) of 23.4Gy and high dose (HD) of 36G. A 0.5 cm clinical target volume margin was used to plan irradiation of the primary site. CSI for low-risk patients were performed using fractionation of 1.5Gy per day; those classified as standard or high-risk were treated with fractions of 1.8Gy per day. Adjuvant chemotherapy was also administered after radiotherapy consisting primarily of four cycles of Cyclophosphamide 1.5 g/m^2^ × 2, Cisplatin 75 mg/m^2^, and Vincristine 1.0 mg/m^2^ × 2. Due to the high-risk of relapse/ progression in the metastatic non-WNT/ non-SHH cohort, three cycles of Pemetrexed 600 mg/m^2^ and Gemcitabine 1250 mg/m^2^ were interspersed with the standard chemotherapy.

MRI scans were performed at four time points: baseline (0M), post six weeks of radiotherapy and six weeks of rest period (3M, 3 months after the baseline), the completion of chemotherapy (12M, 12 months after the baseline), and during follow-up (18–21 months after the baseline). Detailed patient enrollment and inclusion information can be found in Table 1. A total of 273 patients were enrolled in the study. The exams affected by artifacts from motions and metals were excluded from analysis. In addition, patients with large leptomeningeal metastasis were also excluded. As a result, 778 exams were investigated from 241 patients.

**Table 1:** Patient enrollment and inclusion information.

Patients	0M	3M	12M	FollowUp	Total
Drop-off^1^	20	38	57	83	198
Metal & Mets^2^	40	23	15	26	104
Motion	3	3	3	3	12
Evaluable	210	209	198	161	778

1. Drop-off: Patients were dropped off from the study due to death, progressive disease, declining to consent, or not participating at the time points. 2. Metal & Mets: severe artifacts caused by metal or leptomeningeal metastasis in brains.

MRI scans were performed using a 3.0T Siemens whole-body MRI system (Siemens Healthiness, Erlangen, Germany). The imaging protocols were approved by the institutional review board of St. Jude Children’s Research Hospital according to federal regulations and IRB policy. Written informed consent was obtained from patients above 18 years of age and from parents or guardian for patients under 18. An age-appropriate assent process is also followed for patients under 18 years of age. Among the total of 778 exams, 450 were performed under anesthesia from 183 patients, and 328 were conducted without anesthesia from 129 patients. Additionally, CSF shunt procedures to alleviate symptoms related to hydrocephalus in 61 patients resulted in 177 exams with a permanent CSF shunt. Anesthesia administrations included intravenous, inhalation, or mixed methods. The most common agents were propofol and fentanyl for intravenous methods and sevoflurane for inhalation methods. It should be noted that a patient could be initially examined with anesthesia at the early time point and without anesthesia at the late time point. Patient demographic information at four TPs is outlined in Table 2.

**Table 2:** Patient demographic information at four time points.

TP	Sex	Radiation Dose			Shunt		Anesthesia	
		HD	SD	RD	No	Yes	No	Yes
		age range (N)	age range (N)	age range (N)	age range (N)	age range (N)	age range (N)	age range (N)
0M	F	3.3–22.0 (25)	4.1–31.4 (36)	5.7–22.0 (15)	3.3–31.4 (61)	4.8–20.7 (14)	8.3–31.4 (22)	3.3–20.8 (53)
	M	3.5–38.8 (70)	3.4–22.8 (53)	5.3–14.8 (11)	3.4–38.8 (107)	3.5–20.5 (27)	10.2–38.8 (33)	3.4–22.1 (101)
3M	F	4.1–23.1 (29)	5.0–32.2 (31)	6.5–22.8 (17)	3.5–31.7 (57)	4.9–14.9 (17)	8.6–31.7 (26)	3.5–14.9 (48)
	M	4.1–39.6 (66)	4.6–23.6 (48)	6.2–14.6 (7)	3.8–39.0 (101)	3.5–20.8 (33)	4.8–39.0 (56)	3.5–17.6 (78)
12M	F	3.5–22.3 (28)	4.4–31.7 (31)	6.0–22.3 (16)	4.1–32.2 (57)	5.5–15.5 (19)	6.5–32.2 (37)	4.1–14.9 (39)
	M	3.5–39.0 (75)	4.0–23.1 (51)	5.6–14.1 (8)	4.3–39.6 (91)	4.1–21.3 (30)	4.1–39.6 (60)	4.3–18.2 (61)
Follow-Up	F	4.9–16.7 (22)	5.6–32.7 (28)	7.1–23.4 (16)	4.9–32.7 (52)	6.9–17.2 (14)	6.8–32.7 (40)	4.9–15.7 (26)
	M	5.1–40.1 (50)	5.1–24.1 (40)	6.8–15.1 (5)	5.1–40.1 (74)	5.1–19.0 (21)	5.4–40.1 (52)	5.1–20.5 (43)

TP: time point; HD: high dose; SD: standard dose; RD: reduced dose; age ranges are in years; N: the number of patients in each subgroup.

3D T1w images with a resolution of 1 × 1 × 1 mm^3^ were acquired using an MPRAGE sequence (TR = 2000 ms; TE = 2.26 ms; flip angle = 15°; inversion time = 1100 ms; field of view = 221 × 191 × 161 mm^3^). White matter masks were first segmented from 3D T1w images using freesurfer [32]. The white matter masks were manually checked and edited, if necessary, by an advanced signal processing technician and a co-author (JOG).

A pipeline for PVS ratio and volume quantification on T1w images was developed as described by Sepehrband et al [11]. The 3D Frangi filter [33], implemented with Quantitative Imaging Toolkit (https://cabeen.io/qitwiki/), was applied to the T1w images for enhancement and detection of tube-like PVS structures. The filter uses eigenvalues and eigenvectors of the Hessian matrix, which is a matrix of second-order partial derivatives of the images, to determine the scale of structures in the image. A vesselness function was built using the eigenvalues to enhance the PVS structures to generate filtered images which represent the likelihood of a voxel belonging to a PVS. The filtered images were then thresholded to separate the PVS from the surrounding tissue, and then used morphological operations to clean up the segmentation in the segmented perivascular space. Due to the exclusive presence of PVS within white matters, the Frangi filter was selectively applied solely to these specific areas. Frangi filter parameters of α = 0.5, β = 0.5 and c = 2 were used as recommended by Frangi et al [33]. Ventriculostomy catheters, which appeared as dark tubes in the brain, were placed for certain patients undergoing CSF shunts procedures. Leukoencephalopathy, characterized by T1w hypointensity in the white matter, was seen in some patients. To exclude these hypointensities, the maximum PVS size was restricted to 150 voxels to minimize the risk of false positive detection. The PVS segmentation was achieved by applying a threshold of 0.05. Small island structures (<5 voxels) were excluded from the segmentation to minimize noise contribution. The threshold, the maximum and minimum voxels were initially optimized using 40 typical exams by examining PVS mask overlays on T1w images. PVS segmentation was then performed using these parameters. To validate parameter selection, outliers beyond the mean ± 2 * STD(standard deviation) range, along with 20 randomly selected exams within the range, were manually reviewed.

Although the T1w images were collected under the same protocol, the dynamic signal ranges of the images may vary across all the images due to different scanners used, different head positions in the coils/scanners, subject differences, and other physiology and measurement conditions. These intensity variations between the subjects could affect the likelihoods of voxels to PVS because a single fixed threshold is used for all the subjects. Errors could be caused because a single fixed threshold is used for all the subjects. Therefore, the T1w images were normalized before applying the Frangi filter, using a fuzzy c-mean (FCM-based) normalization method (https://github.com/jcreinhold/intensity-normalization) [34] to a mean value of 110 in white matter. The PVS quantification method using T1w images only was validated by comparing with established T1w/T2w method [11] (see S1 Appendix in Support information).

### Full model: All data

For all 778 exams, we compared the PVS (including the PVS volume and the PVS ratio which is the ratio of PVS volume to white matter volume in ‰) at four time points using statistical analyses. First, we examined the influence of time point (TP), irradiation dose, anesthesia, age, sex, and shunt procedure on the PVS using a mixed model. This model was chosen because it can effectively accommodate repeated measurements, which constituted most of our data. The mixed model allowed us to account for the correlated data within the same subject. The mixed model incorporated all variables to assess their impact on PVS.


$$PVS=\beta_{0}+\beta_{1}TP+\beta_{2}\text{Anesthesia}+\beta_{3}Age+\beta_{4}Sex+\beta_{5}DOSE+\beta_{6}Shunt$$ (1)

where *β*_*0*_ is the intercept, and *β*_*i*_ (*i* = 1,2,3,4,5,6) are the effect estimates associated with TP, Anesthesia, Age, Sex, DOSE, and Shunt respectively. All variables were treated as fixed effects. The Bonferroni method was used for multiple testing adjustment, and the p values after the adjustment are represented as p_adj.

### Paired model: Paired data between consecutive time points

Given the involvement of six variables in the full model, we aimed to isolate certain variables, such as anesthesia, shunt, age, and sex, to narrow our focus on examining the impact of radiotherapy and chemotherapy on PVS. Specifically, for consecutive time points, such as baseline and 3M during the radiotherapy period, we only selected patients who completed both measurements under the same anesthesia and shunt conditions. Given small time differences and the gradual nature of age-related effects on PVS, we could consider age as having minimal influence or as a non-dominating factor, especially during the radiotherapy period (0M–3M). Thus, our primary focus rested on discerning treatment and recovery effects on different dose levels within this context. The PVS differences, denoted as ΔPVS_ratio or ΔPVS_volume, between two consecutive measurements for each patient were computed. A one sample T-test was performed to test whether ΔPVSs (including ΔPVS_ratios and ΔPVS volume) were significantly different from zero, that is, whether PVSs significantly increase or decrease between two TPs. In addition, varying levels of radiation dose might affect the PVS changes differently, and the changes at different dose levels were statistically compared using ANOVA (Analysis of Variance). The data analysis in this study was generated using SAS 9.4 software (SAS Institute Inc., Cary, NC, USA).

## Results

**Fig 1:**
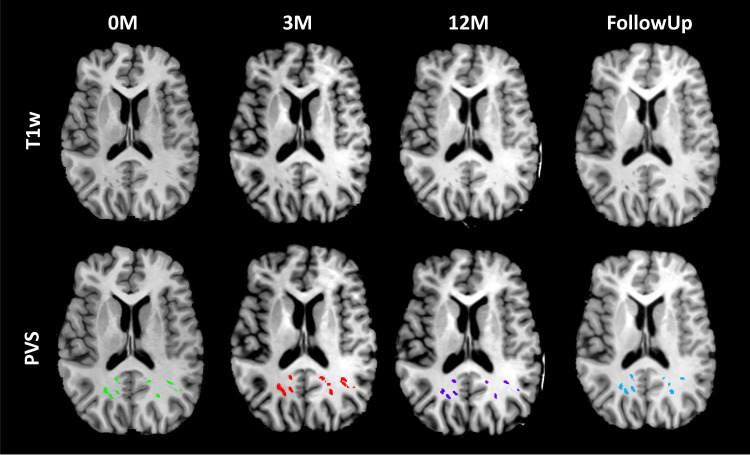
T1w images (top row) and segmented PVS overlaid on T1w images (bottom row) at TP of 0M, 3M, 12M, and FollowUp for a typical patient.

Fig 1 shows T1w images of a patient at four TPs (the top row), along with the PVS segmentations overlaid on the corresponding T1w images (the bottom row). All images and PVS masks were co-registered to the baseline T1w images. This figure illustrates that the PVS were effectively identified by the software.

### Full model: All data

**Fig 2:**
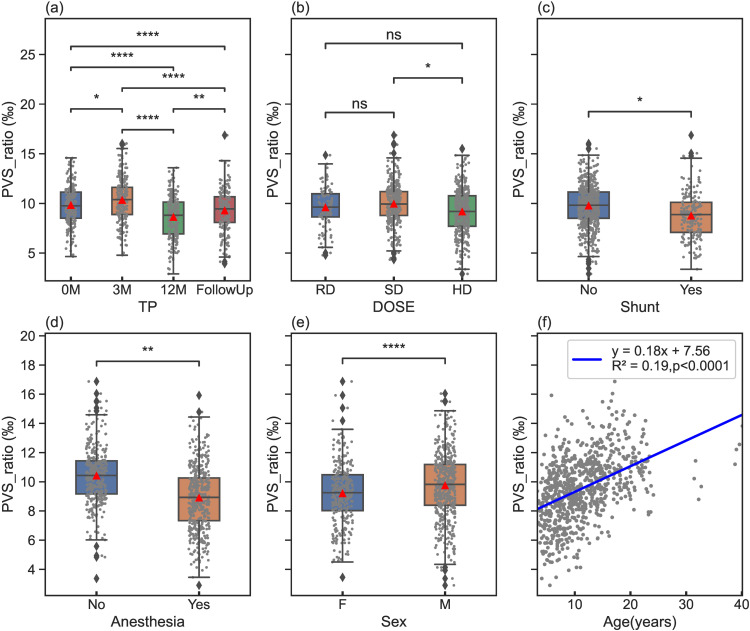
PVS ratio comparisons across different variables using box plots with statistical comparison annotations for TP(a), DOSE(b), Shunt(c), Anesthesia(d), and Sex(e). Statistical significance levels are annotated on the box plots, denoted as follows: ns(not significant): 0.05 < p_adj <= 1; * : 0.01 < p_adj **≤** 0.05; **: 0.001 < p_adj **≤** 0.01; ***: 0.0001 < p_adj **≤** 0.001; ****: p_adj **≤** 0.00001. In addition, a scatter plot (f) illustrates the relationship between PVS ratio and age with a line of best fit and equation. The red triangles indicate the mean values of each sub-group.

A comprehensive statistical model was evaluated, incorporating all variables to assess their impact on PVSs (including PVS ratio and PVS volume) with the model. Fig 2 shows the statistical comparisons for each variable for PVS ratios. The full data set was attached in S2 Dataset1 in Support information. The mean of PVS ratios increased from 0M to 3M, decreased from 3M to 12M, and increased again from 12M to FollowUp. The means and standard deviations (STD) were 9.8 ± 1.9‰ for 0M, 10.3 ± 2.2‰ for 3M, 8.6 ± 2.1‰ for 12M, and 9.3 ± 2.1‰ for FollowUp. The means ± STD of the ratios were 9.6 ± 2.0‰, 9.9 ± 2.1‰ and 9.2 ± 2.3‰ for DOSE level RD,SD and HD, respectively. The effect of different dose levels was not significant different except between SD and HD as shown in Fig 2b. Fig 2c shows that patients who had undergone a shunt procedure exhibit a significantly reduced PVS ratio (8.7 ± 2.2‰) compared to those (9.8 ± 2.1‰) who had not undergone the procedure. Fig 2d indicates that the PVS ratios obtained under anesthesia (9.9 ± 2.1‰) were significantly lower than those without anesthesia(10.4 ± 1.9‰). Additionally, females exhibit a significantly lower PVS ratio (9.1 ± 2.1‰) than males (9.7 ± 2.2‰) as shown in Fig 2e. An increase of PVS ratio with age at a slope of 0.18‰/year was also demonstrated in Fig 2f.

**Fig 3:**
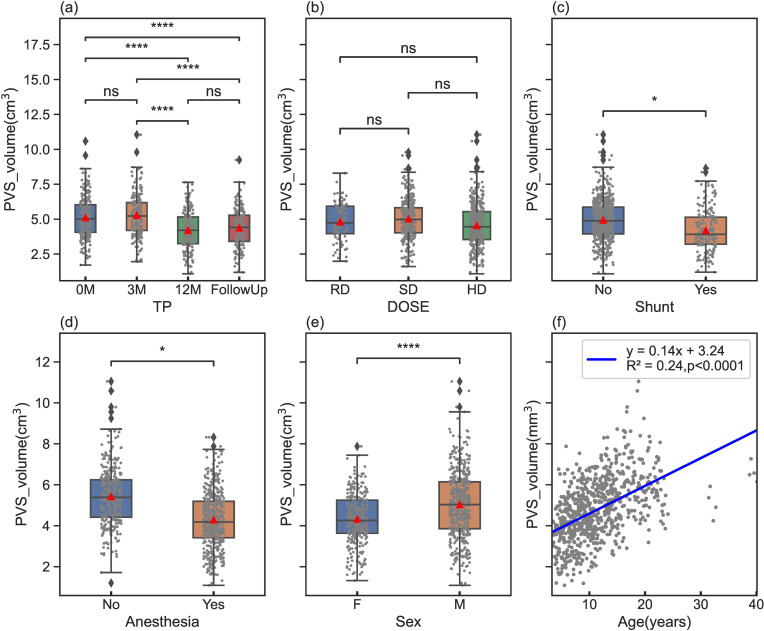
Box plots representing comparisons of PVS volumes across different variables with statistical comparison annotations for TP(a), DOSE(b), Shunt(c), Anesthesia(d), and Sex(e). Statistical significance levels are denoted as follows: ns(not significant): ns(not significant): 0.05 < p_adj <= 1; * : 0.01 < p_adj **≤** 0.05; **: 0.001 < p_adj **≤** 0.01; ***: 0.0001 < p_adj **≤** 0.001; ****: p_adj **≤** 0.00001. Additionally, a scatter plot (f) demonstrates the relationship between PVS volume and age with a line of best fit and equation. The red triangles indicate the mean values of each sub-group.

Similar findings were observed in the PVS volume, and the statistical comparisons are included in Fig 3. The changing trends in PVS volumes followed the same pattern as the PVS ratios, with significance levels largely consistent between the two measures. However, there were a few exceptions: no significant differences were found between 0M and 3M, as well as between 12M and FollowUp on TPs (Fig 3a); additionally, no significant differences were observed between different dose levels (Fig 3b). The figure further revealed that PVS volume exhibited a positive correlation with age, increasing at a rate of 0.14 cm^3^ per year (Fig 3f).

### Paired model: Paired data between consecutive time points

**Fig 4:**
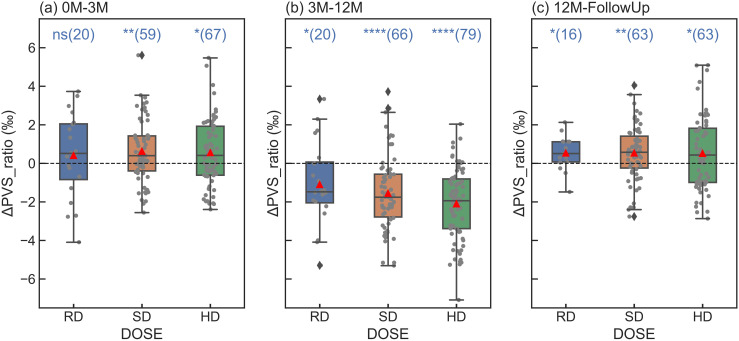
PVS_ratio changes, ΔPVS_ratio, for different radiation doses within each treatment period: 0M–3M(a), 3M–12M(b), and 12M–FollowUp(c). The statistical annotations in the figures indicate results from T-test examining whether **Δ**PVS_ratios significantly differ from zero. The numbers inside the parentheses are the numbers of patients in each sub-group. Statistical significance levels are denoted as follows: ns(not significant): 0.05 < p <= 1; * : 0.01 < p **≤** 0.05; **: 0.001 < p **≤** 0.01; ****: p **≤** 0.00001. The red triangles indicate the mean values of each sub-group.

We examined the changes in ΔPVSs (including ΔPVS_raio and ΔPVS_volume) of the patients who completed two consecutive time points under the same conditions of shunt and anesthesia. Fig 4 illustrates the PVS ratio changes at different treatment periods, (a) 0M–3M, (b) 3M–12M, (c) 12M–FollowUp, for different DOSE levels (RD, SD and HD), along with statistical annotations. The full data set of this comparison was included as S3 Dataset2 in Support information. The means of ΔPVS_ratios were positive for the radiotherapy period (0–3M) as shown in Fig 4a, negative for the chemotherapy period (Fig 4b, 3M–12M), and positive again for the recovery phase (Fig 4c, 12M–FollowUp). All those changes were significant except for RD at 0M–3M as shown in Fig 4a. These findings further confirmed the observations made with the full model. Across each duration, variations in mean ΔPVS_ratios were noticeable for different dose levels. However, no statistically significant differences were observed among them.

**Fig 5:**
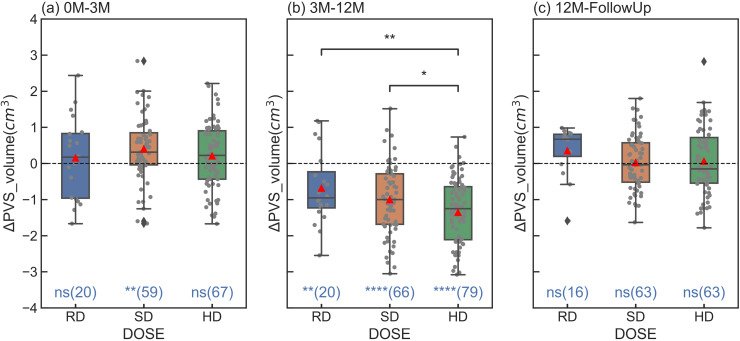
PVS_volume changes, ΔPVS_volumes, for various radiation doses within each treatment period: 0M–3M(a), 3M–12M(b), and 12M–FollowUp(c). The statistical annotations on the bottom in the figures indicate results from T-test assessing whether **Δ**PVS_volumes significantly differ from zero. The numbers inside the parentheses are the numbers of patients in each sub-groups. Significant differences were observed between RD and HD, as well as between SD and HD. However, no significant differences were found between other dose levels (not explicitly labelled). Statistical significance levels are denoted as follows: ns(not significant): 0.05 < p <= 1; * : 0.01 < p **≤** 0.05; **: 0.001 < p **≤** 0.01; ****: p **≤** 0.00001. The red triangles indicate the mean values of each sub-group.

Similar results were also obtained regarding the changes of PVS volume, and the statistical comparisons were included in Fig 5. Compared to the ratios, the changes of the volumes shared the same changing pattern with the ratios, with significance levels largely consistent between the two measures, except that significant differences were found between RD and HD, as well as SD and HD (Fig 5b). Within 12M–FollowUp period no significant differences from zero were found for all dose levels (Fig 5c). Table 3 lists the means and standard deviations of ΔPVS_ratios and ΔPVS_volumes for different dose levels at different durations.

**Table 3:** Changes of PVS ratios and volumes for different treatment periods and different dose levels (HD: high dose, SD: standard dose, and RD: reduced dose).

Period	ΔPVS_ratio(‰)			ΔPVS_volume(cm^3^)		
	RD	SD	HD	RD	SD	HD
0M–3M	0.41 ± 2.19	0.63 ± 1.66	0.55 ± 1.75	0.15 ± 1.11	0.40 ± 0.90	0.21 ± 0.91
3M–12M	−1.09 ± 2.1	−1.55 ± 1.90	−2.09 ± 1.81	−0.68 ± 1.02	−1.00 ± 0.97	−1.35 ± 1.00
12M–FollowUp	0.54 ± 0.88	0.53 ± 1.44	0.52 ± 1.92	0.35 ± 0.68	0.02 ± 0.78	0.06 ± 0.94

## Discussion

To the best of our knowledge, this is the first systematic investigation into the influence of radiotherapy and chemotherapy on PVS. By analyzing PVS changes across four unique time points during the treatment, this study helps us for a better understanding of the impact of treatment on perivascular spaces. Moreover, this study reported the first systematic examination of the effects of shunt and anesthesia on PVS, a crucial step for the potential clinical application of PVS. Both PVS ratios and PVS volumes were investigated in this study, yielding generally the same findings. However, it’s worth noting that they are not entirely identical; rather, they exhibit a degree of complementarity, because the sizes of white matters might vary among the patients. The patient cohort under study is distinctive, with approximately one-third of the patients being under the age of eight.

Through both the full model and paired model, our study shows that PVS ratio is significantly altered after radiotherapy, with the PVS ratio increasing during irradiation. We also observed a significant decrease in the PVS ratio after irradiation and during chemotherapy. However, at FollowUp an increase in the PVS ratio was observed. The rise in PVS due to irradiation may be attributed to the disruption of the blood-brain barrier(BBB) induced by the irradiation. It is widely acknowledged that radiotherapy can elevate permeability and lead to BBB disruption [25,35]. Studies have observed an increase in BBB permeability following radiotherapy [36]. This breakdown of the BBB results in fluid leakage into the PVS, and BBB breakdown may be part of the pathogenesis of enlarged PVS [25,26,37]. Upon completion of irradiation, the BBB would naturally undergo recovery from the damage, with permeability levels gradually returning to those seen before treatment over a period of eight months [36]. At this stage, a reduction in PVS would be typically anticipated due to the recovery process. However, in this study, the influence of chemotherapy complicates this interpretation. The observed reduction in PVS could also be impacted by the effects of chemotherapy on top of the recovery. From 12M to FollowUp a PVS rise was observed, with a significant difference in the PVS ratio but not in the PVS volume with both the full model and paired model. The persistent effects of the previous radiation and chemotherapy regimens might have led to a gradual increase in PVS burden over time, despite the treatments having concluded.

As the degree of destructive effect on the BBB was directly proportional to the radiation dose [36], we expected that the radiation-induced PVS changes to be related to varying radiation dose levels. Our observations generally support these conclusions. We noted that the lowest radiation dose (RD) resulted in the smallest increase in PVS ratios and volumes. However, the highest radiation dose (HD) did not lead to the largest increase in PVS changes. Furthermore, none of these changes were statistically significant.

Large or giant PVS volumes were first reported for adults following irradiation. Giant PVS volumes following irradiation in a 45 year old patient was first reported as a clinical finding in 2018 [28]. In another clinical report, Mark et. al. reported enlarged PVS after radiotherapy with a total dose of 54Gy in two adult patients (50 and 46 years old) with medulloblastoma [29]. Recently Veiga et al manually examined 139 pediatric patients for cyst-like lesions as a late sequela of radiotherapy, and they considered the lesions as enlarged PVS [38]. Visual inspection was the commonly applied method in all the aforementioned mentioned studies. Our study further confirmed these findings quantitatively in a cohort of 241 pediatric patients at four time points using automatic Frangi filter method, and we systematically verified the enhancement of PVS by radiotherapy. We did not observe any giant PVS volumes as reported in adults. This discrepancy may be attributed to the predominantly young age of the pediatric patients in our study.

Shunt surgery is intended to drain excess CSF from the brain’s ventricles. Patients who underwent CSF diversion and placement of a permanent CSF shunt exhibited lower PVS ratios. This may be indicative of glymphatic function recovery following shunt surgery. A previous study reported an increase in the DTI-ALPS index after the shunt procedure [39]. However, the DTI-ALPS index was found to be negatively correlated with the PVS score, and they often played reversed roles for diseased states [22,23]. This evidence supports our findings that shunt surgery decreases PVS.

Anesthesia is commonly administered to pediatric patients due to the difficulty of remaining motionless during lengthy MRI exams. Anesthesia leads to a reduction in the cerebral metabolic rate for oxygen, which in turn decreases cerebral blood flow (CBF) since these two factors are closely coupled [40]. Furthermore, the CSF flow was significantly reduced under anesthesia in mice [41]. The decrease in CBF and CSF flow may account for the observed reduction in PVS ratio during exams conducted under anesthesia. Therefore, it is crucial to consider the potential impact of anesthesia on PVS changes when incorporating these measures in a study.

In addition, this study also confirmed a few findings which were reported previously. Males had a significantly higher PVS ratio than females [42]. The PVS increases with age which is in agreement with a previous finding [20,42]. In additional to age and sex, the PVS ratios are influenced by a range of factors, including anesthesia, and shunt procedures as shown in this study. Utilizing a mixed effect model, we sought to scrutinize the influence of the covariate of interest, such as TP, while adjusting for the effects of other covariates like age, sex, shunt, and dose level. The mixed model with the repeated measurements handles the correlations of PVS ratio within the patients. Furthermore, we simplified the comparison by selecting a paired subgroup from the whole cohort and examined the changes of PVS ratios in each treatment period. Given the conditions of shunt, anesthesia and sex were matched for the consecutive TPs, any observed changes were primarily attributed to the treatment and/or recovery process. Considering PVS ratios tend to increase with age, the decrease observed during 3M–12M period couldn’t be attributed to the age. It is noteworthy that both the full model and the paired model reach the same conclusions and confirm each other. Furthermore, the observations in this study find direct or indirect support in prior research as discussed above. The impacts of radiation, sex, age, shunt, anesthesia on the PVS are all in agreement with the previous observations [28,42,43]. These collective findings affirm the appropriateness of the methodologies employed in this study.

PVS, as part of the glymphatic system, serves as the primary channel for draining interstitial fluid (ISF) from brain tissues. It plays a crucial role in facilitating the exchange between CSF and ISF, cleaning intracerebaral waste, and maintaining brain homeostasis. Additionally, the PVS serves as a pathway for transporting various signaling molecules and metabolic factors [44]. Dysfunction in the glymphatic system has the potential to cause the enlargement of the perivascular space (PVS) and the accumulation of misfolded protein aggregates. An enlarged PVS load may be an indicator of compromised endothelial walls and disrupted exchange of ISF, subsequently contributing to glymphatic dysfunction [45]. Consequently, evaluating changes in the PVS could be employed to gauge the impact of radiotherapy toxicity and neurological conditions on patients.

One limitation of this study was the relatively small number of patients in the lowest reduced dose (RD) group. A more substantial number of patients across various radiation dose levels is necessary to establish a more robust statistical inference on how the radiation dose level affects PVS ratios. The impact of the resection surgery was not investigated because no MRI data were collected before the resection in this retrospective study.

The immediate effects of radiotherapy seem to lead to an elevated PVS, suggesting acute damage on BBB. However, during chemotherapy in the period post- irradiation, the PVS decreases, possibly indicating a recovery combined with the effect of radiotherapy and chemotherapy. At the FollowUp time point, post-chemotherapy, the PVS ratio again increased. This increase may be the start of more chronic effects of radiotherapy and chemotherapy.

## Supporting information

S1 Appendix Validation of PVS quantification using T1 weighted images. (DOCX)

S2 Dataset1 The dataset for the full model analysis. The spreadsheet includes age, sex, TP, DOSE, anesthesia, shunt, pvs_ratio (‰) and pvs_volume (cm^3^) information for all patients.
(XLSX)

S3 Dataset2 The dataset for the paired model. The spreadsheet includes Δpvs_ratio (‰) and Δpvs_volume (cm^3^) information.
(XLSX)

## Abbreviations

PVS: perivascular Space
CSF: cerebrospinal fluid
TP: time point
MB: medulloblastoma
T1w: T1 weighted
T2w: T2 weighted
CBF: Cerebral Blood Flow
BBB: Blood-Brain Barrier
