# Supplementary material for: Perivascular space imaging during therapy for medulloblastoma
Source: PLoS One. 2025 Feb 7;20(2):e0318278. doi: 10.1371/journal.pone.0318278 (PMC11805390; doi:10.1371/journal.pone.0318278)
Supplement: S1 Appendix — (DOCX) [file pone.0318278.s001.docx]

Support information, Appendix

Validation of PVS quantification using T1 weighted images

T1w method was validated by comparing with the established combined T1w/T2w method introduced in reference 11. T1w and T2w images of 198 pediatric subjects (age:8-18 years, Female=Male=99) were obtained from the Human Connectome Project (HCP, https://www.humanconnectome.org/ ). PVS was segmented in T1w, T1w/T2w images following the same procedure detailed in the Methods section of this manuscript.


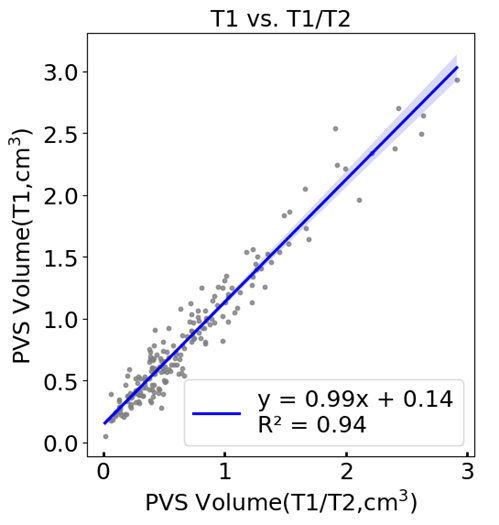


**Fig S1. Scatter plots showing the relationship between PVS volumes measured using T1w and T1w/T2w images.**

Fig S1 illustrates the relationships between PVS volumes from T1w and T1w/T2w images. Strong correlations were observed (R² = 0.94).


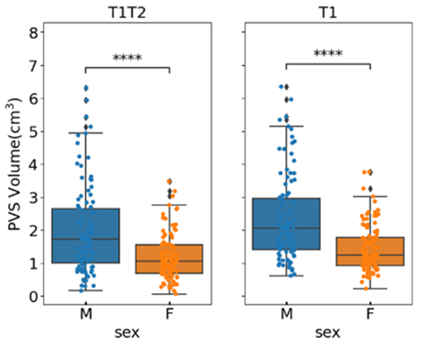


**Fig S2. Comparison of PVS volume between males and females across T1 and T1/T2 methods**

Fig S2 shows PVS volumes by sex across T1w and T1w/T2w methods, and both show significantly higher PVS ratios in males with p < 0.0001.

Test-retest data include 45 subjects (Female=32) from the HCP S1200 project. The test-retest subjects were scanned twice using the same scan protocol with a mean interval of about 140 days.


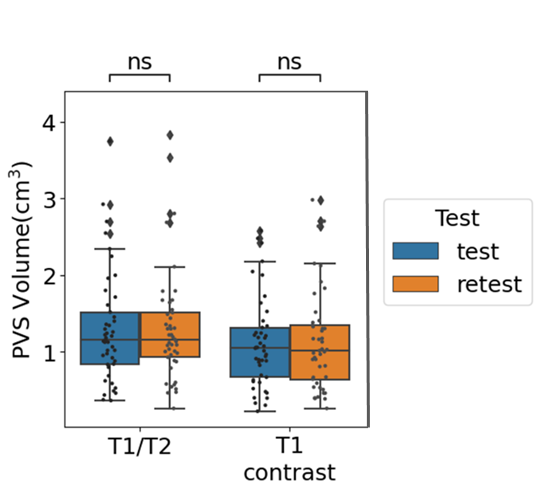


**Fig S3. Test-retest reliability of PVS volume measurements across T1w and T1w/T2w methods. Boxplots show PVS volume for test (blue) and retest (orange) sessions.**

Figure S3 demonstrates method repeatability, showing no significant differences between test and retest for all methods.

By demonstrating the correlation, the capability to detect the PVS difference between male and female, and test-retest performance, T1w method can be used to quantify PVS.
